# The thymus road to a T cell: migration, selection, and atrophy

**DOI:** 10.3389/fimmu.2024.1443910

**Published:** 2024-08-27

**Authors:** Mario Ruiz Pérez, Peter Vandenabeele, Peter Tougaard

**Affiliations:** ^1^ Molecular Signaling and Cell Death Unit, VIB-UGent, Center for Inflammation Research, Flanders Institute for Biotechnology, Ghent, Belgium; ^2^ Department of Biomedical Molecular Biology, Ghent University, Ghent, Belgium; ^3^ Laboratory of Immunoregulation and Mucosal Immunology, VIB-UGent Center for Inflammation Research, Ghent, Belgium

**Keywords:** thymus organogenesis, thymus morphology, thymus colonization, T cell development, acute thymus atrophy, thymocyte cell death

## Abstract

The thymus plays a pivotal role in generating a highly-diverse repertoire of T lymphocytes while preventing autoimmunity. Thymus seeding progenitors (TSPs) are a heterogeneous group of multipotent progenitors that migrate to the thymus via CCR7 and CCR9 receptors. While NOTCH guides thymus progenitors toward T cell fate, the absence or disruption of NOTCH signaling renders the thymus microenvironment permissive to other cell fates. Following T cell commitment, developing T cells undergo multiple selection checkpoints by engaging with the extracellular matrix, and interacting with thymic epithelial cells (TECs) and other immune subsets across the different compartments of the thymus. The different selection checkpoints assess the T cell receptor (TCR) performance, with failure resulting in either repurposing (agonist selection), or cell death. Additionally, environmental cues such as inflammation and endocrine signaling induce acute thymus atrophy, contributing to the demise of most developing T cells during thymic selection. We discuss the occurrence of acute thymus atrophy in response to systemic inflammation. The thymus demonstrates high plasticity, shaping inflammation by abrogating T cell development and undergoing profound structural changes, and facilitating regeneration and restoration of T cell development once inflammation is resolved. Despite the challenges, thymic selection ensures a highly diverse T cell repertoire capable of discerning between self and non-self antigens, ultimately egressing to secondary lymphoid organs where they complete their maturation and exert their functions.

## 1 Introduction

### 1.1 A short history of the thymus

The term *“thymus”*, derived from the ancient Greek *“θυμός (thumos)”*, meaning *“soul/spirit”*, possibly believed to be the soul’s dwelling place for its central location just above the heart. For centuries the function of the thymus remained enigmatic, and until the 1950 no apparent immune function could be attributed to the thymus due to; 1) the apparent absence of health problems in thymectomized adult individuals 2) the lack of germinal centers as compared with the spleen, 2) the lack of antibody-producing cells post-immunization, 4) the insufficient immune responses following thymus lymphocyte transfer to immunocompromised recipients, 4) the misconception about the reduced thymus size in individuals that succumbed to infectious diseases versus young healthy individuals (1, 2).

It wasn’t until a series of pioneering studies by Jaques Miller and colleagues between 1959 and 1957 that the thymus function was elucidated, the last among the major internal organs to be discovered (3–7). Performing either thymectomy or bursectomy in chickens Miller’s team demonstrated the thymus’s essential role in cellular immunity and, in conjunction with the bursa for the cellular and humoral responses (5, 7–9). Despite these experiments and firm conclusions, one of the leading immunologists of that period, Peter Medawar (1915 – 1987) stated: *“We shall come to regard the presence of lymphocytes in the thymus as an evolutionary accident of no very great significance”* (10). Nevertheless, Miller’s discoveries contributed to enduring paradigms in immunology still valid to this day: 1) The thymus is not only a source for T cells but also the site where T cell progenitors are “trained” for self-tolerance and develop immunocompetence through interactions with thymic epithelial cells and other professional antigen-presenting cells (APCs); 2) Due to the role of T cells mediating allograft rejection, repressing T cells is a valuable therapeutic approach for transplantation; 3) B and T cells are different lineages that adopt their fate in different organs and; 4) Effective immune response requires a collaboration from both B and T cells. These paradigms laid the foundation of T cell biology, profoundly impacting modern medicine in fields like autoimmune and inflammatory diseases, oncology, and organ transplantation.

### 1.2 Thymus origin in evolution

The thymus is evolutionary conserved across species in jawed vertebrates (gnathostomes) (11). The anatomical position, the number of thymic lobes per animal, and its organogenesis can vary between species (12). The presence of a thymus-like organ has been suggested in the cyclostomes (lampreys) (13). Nevertheless, *Chondrichthyes* (cartilaginous fish, i.e. sharks and rays) are considered the oldest thymus-bearing taxon (12). Parts of the genetic network underlying thymopoiesis was already present before the thymus originated (14) from an ancient gut-associated lymphoid tissue (GALT) (12, 15). The prevailing view is that as the cellular immune system developed, the thymus evolved in relation to its role in immune tolerance to cope with the increased potential for self-reactive T cells (16). As the evolutionary origin of the thymus has been linked to the evolution of jaws in the cartilaginous fishes, its origin might also be related to their wider access to food sources increasing the need of a more specific immune responses (16).

### 1.3 Thymus organogenesis

The thymic organogenesis differs across species in terms of timing and the number of pharyngeal pouches required for its development (12). In mammals, thymus organogenesis is a multi-step process involving 1) thymus fate determination from the common thymus-parathyroid primordium, 2) detachment from the parathyroid, 3) migration to the thorax cavity and subsequently, 4) multipotent progenitors (MPP) colonization. In humans, the thymus continues growing during postnatal development until reaching its peak in cellularity few weeks after birth, followed by a decline starting before puberty (17). These kinetics are similar in mice, humans, equines, and zebrafish (18). The thymus derives from the pharyngeal pouches of the endodermal gut tube around embryonic day 9.5 (E9.5, “E#” hereafter referring to day of murine embryonic development), in a process regulated by HOX3, PAX1, PAX9, FGF8, and FOXN1 (the earliest thymus-specific marker, detected at E11) (15). By E11.5, the third pharyngeal pouch has formed the thymus and parathyroid structures, preparing for their separation, guided by the *Pax-Eya-Six* gene cascade. Surrounded by a mesenchymal capsule from neural crest cells, these structures then detach from the pharynx around E12.5 and begin migrating to the anterior thoracic cavity (19). The dissociation of the thymus from the parathyroid, potentially influenced by FOXN1 and GCM2, is followed by the thymus’s outgrowth regulated by HOX and FGF family members (19, 20). By E15 (8-10 weeks of gestation in humans) (21, 22), the thymus is positioned in its final anatomical location, coinciding with the colonization of hematopoietic precursors necessary for lymphopoiesis (12, 23).

The vasculature and lymphatic systems are developed in parallel in the embryo. The blood vascular system is one of the first functional systems formed in the body during embryonic development (starting from E8.5) (24) while the lymphatic system is initiated from day E9.5-10.5 (25, 26). The thymus vascularization starts around E15, while the onset of the lymphatic wiring remains unknown (27). Unlike other lymphoid organs, the thymus does not contain afferent lymphatic vessels entering it. It only contains efferent lymphatic vessels from where thymocytes egress to the periphery (28). Notably, the migration of multipotent progenitors (MPPs) from the different niches (yolk sac, fetal liver, and bone marrow) to the thymus starts prior formation of vasculature and lymphatic system (29). This process is crucial as a first step of T-cell development and it will be discussed in the coming sections.

## 2 The thymic landscape: architecture and compartments

**Figure 1:**
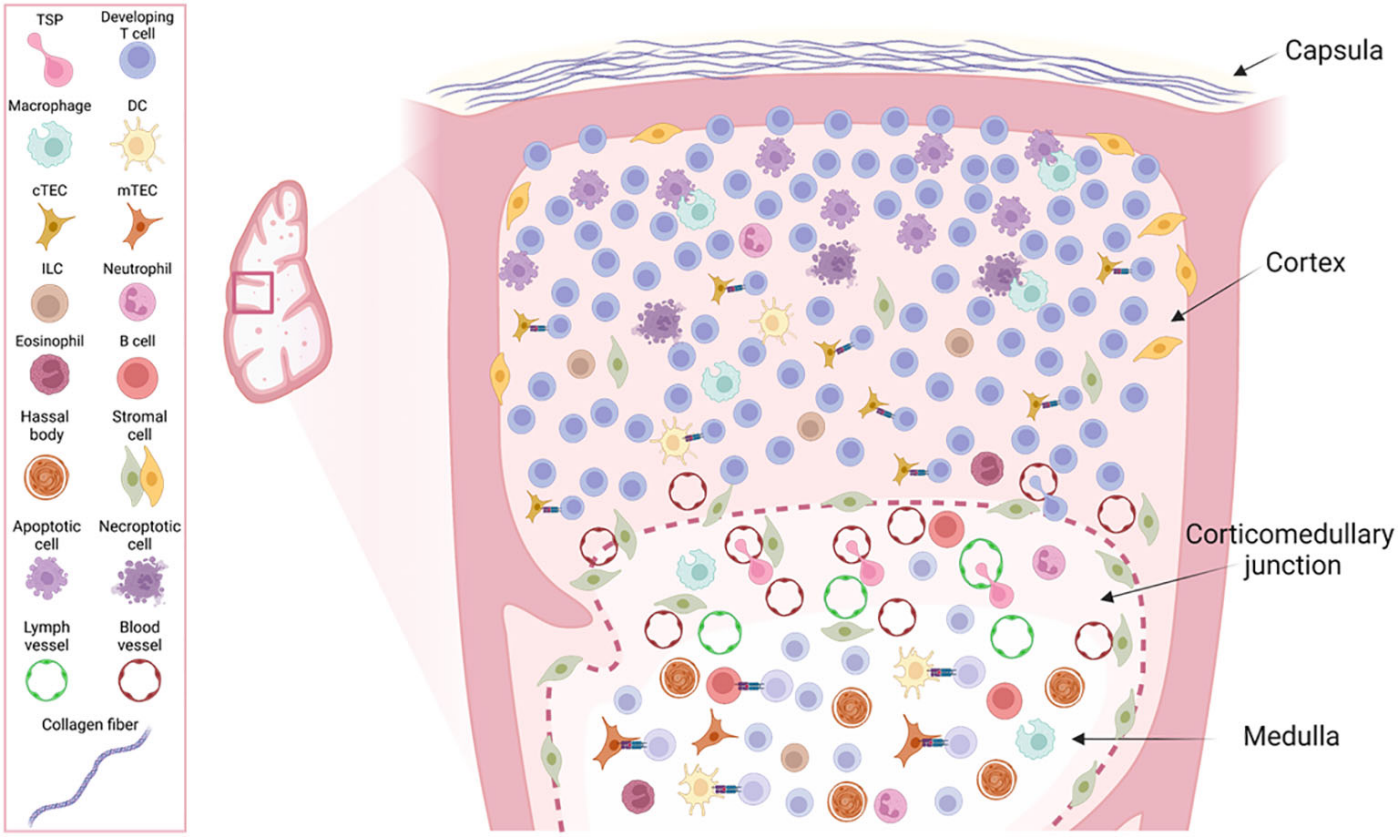
Overview of the structural layers and cellular composition of the thymus lobe. The figure illustrates a zoom view of the four different morphological layers from the outer to the inner: capsula, cortex, corticomedullary junction (CMJ) and medulla and the diversity of cell types contained within those layers with their reported anatomical localization within the thymus. The schematic does not represent their relative numbers within the thymus tissue.

The compartmentalized architecture of the thymus is essential for allowing the correct development and selection of T cells. In mammals, the thymus is a bi-lobulated organ separated by an interlobular septum and linked by connective tissue. The thymus morphology and cellular composition is highly conserved across species (30). Its microanatomy comprises connective tissue, extracellular matrix components, epithelial cells, and various immune cells. The immune cell compartment is predominantly formed by developing T lymphocytes, but also includes minor populations of dendritic cells (DCs), macrophages, monocytes, neutrophils, eosinophils, natural killer (NK) cells, innate-lymphoid cells (ILCs), and B cells (31–36). Structurally, the thymus is organized into four distinct morphological and histological layers: the capsula, cortex, corticomedullary junction, and medulla, arranged from the outermost to the innermost layer (37), as illustrated in **Figure 1**.

### 2.1 Capsula

The thymus is encased in a connective tissue layer known as *“capsula”*, which envelops both lobes. The capsula comprises an outer and inner layer made up of collagen and reticular fibers. The inner layer can invaginate to form septa, from which thin trabeculae that contain vascularized capillaries extend towards the center of the thymus lobe. Approximately 5% of the developing thymocytes are found in the subcapsular area, region proximate to the outer cortex (21).

### 2.2 Cortex

The cortex is the next distinct histological region of the thymus, characterized by a high concentration of immature lymphocytes which outnumber epithelial cells, macrophages, and dendritic cells that support T-cell development. Noteworthy, there is a gradient of less mitotically active cells towards the inner part of the thymus getting close to the corticomedullary junction, that reflects the different stages of the development of pre-T cells, directly correlated with their anatomical position within the thymus (21). The cortex is the most cellular dense region in the thymus, accounting for about 70-80% of them, and is the primary site of positive selection (21). The cortex harbors the cortical thymic epithelial cells (cTECs) that play a role in the positive selection of developing thymocytes.

### 2.3 Corticomedullary junction

The inner cortex borders the corticomedullary junction, a layer characterized by a high density of blood and lymph vessels, supported by connective tissue (23). The thymus only contains efferent lymphatic vessels from where thymocytes egress into circulation. As a consequence, unlike lymph nodes, the thymus does not swell during an infection (28). Arteries supply the organ through the corticomedullary region, branching into capillaries that extend into the medulla and cortex. Generally, cortical capillaries are less fenestrated, resulting in limited circulation of antigens, whereas medullary capillaries are fenestrated, facilitating antigen flow (38). Nerve fibers innervate the thymus, following the vasculature within the capsule and septum adjacent to the corticomedullary junction (38). The areas surrounding large blood vessels in the CMJ are called perivascular spaces (PVS) (39). PVS primarily contain recently infiltrated early thymic progenitors (ETPs), SP CD4^+^, and SP CD8^+^ cells, but also plasma cells are present in the PSV (40, 41). Thymus seeding progenitors (TSPs) enter the thymus through large venules located at the corticomedullary junction and once they become naïve CD4^+^ or CD8^+^ T cells re-enter the circulation through the post-capillary venules. This area thus becomes a hub for both incoming immature lymphocytes and mature lymphocytes preparing to exit the thymus (23).

### 2.4 Medulla

The medulla forms the central layer of the thymus and continues between adjacent lobules, often extending deep into the cortex, near the capsular region. This region, less dense than the cortex, contains mature T cells, epithelial cells, Hassall’s corpuscles, macrophages, dendritic cells, B cells, and other myeloid cells (23). Approximately 10-15% of all thymocytes, predominantly single positive (SP) CD4^+^ or CD8^+^, are located in the medullary region, undergoing negative selection (Expanded in Section 3.2). The thymus medulla hosts various antigen-presenting cells (APCs) including medullary thymic epithelial cells (mTECs), DCs, macrophages, and B cells, which present self-peptides via major histocompatibility complexes I (MHC-I) and II (MHC-II) (42). The affinity between TCR and MHC-self peptide is a crucial determinant for the selection fate of developing thymocytes (expanded in Section 3.2). Those that have strong TCR affinity are negatively selected by programmed cell death (PCD). Thymocytes that successfully overcome the positive and negative selection steps, become mature and can eventually egress from the thymic medulla blood vessels into circulation. Localizing in the medulla, Hassall’s corpuscles, are thought to be involved in the clearance of dying thymocytes (43), and are an important source of thymic stromal lymphopoietin (TSLP) involved in dendritic cell instruction and regulatory T cells (Treg) induction in the thymus (44).

The compartmentalization of the thymus is critical for enabling the correct development and selection of developing T cells as specialized processes occur in different morphological layers that contain diverse immune-, and stromal- cells that orchestrate T cell development and survival. The precise location of each subset of developing T cells in the different structural layers will be described in the following sections.

## 3 How to become a T cell

Functioning as a primary lymphoid organ, the thymus provides the essential microenvironment to support the generation of a highly-diverse and self-tolerant T cell repertoire. The thymus is a dynamic organ that contains a high number of different cell types working together to allow the complex process of T-cell development. T cell development is a well-orchestrated, tightly-regulated process that encompasses a set of steps including: colonization by multipotent progenitors (45, 46), T cell specification, commitment, selection (47–51), and tolerance instruction (52).

### 3.1 Migration of multipotent progenitors to the thymus

**Figure 2:**
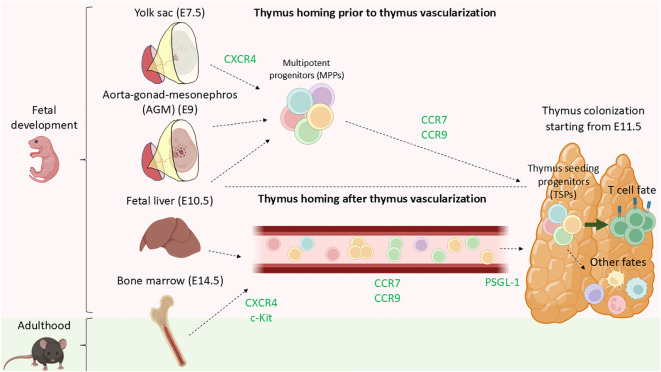
Current model of thymus colonization by MPPs during fetal development and thorough adulthood. During fetal development, CXCR4 plays a role in aiding MMPs being mobilized from either AGM, yolk sac, or fetal liver even prior complete formation of the blood circulation system. MPPs, primarily LMPPs and CLPs migrate to the thymus in a CCR9-, CCR7-dependent manner. Shortly after birth, the bone marrow becomes the primary site of MPPs residency and export. Analogous to during fetal development, progenitors are mobilized into circulation via CXCR4 and c-Kit and later guide towards the thymus by CCL21 and CCL25 (CCR7 and CCR9 ligands) by chemotaxis. Lastly PSGL-1 plays a role in the transmigration of the TSPs for entering the thymus.

Self-renewing hematopoietic stem cells (HSCs) have not been shown in the thymus (53) and therefore, the thymus is believed to rely on continuous seeding of multipotent progenitors (MPPs) from the aorta-gonad mesonephros (AGM), yolk-sac, fetal liver, or fetal bone marrow during embryogenesis and bone marrow throughout adult life (54) (**Figure 2**). Notably, not all circulating MPPs are able to successfully settle in the thymus. Thymus-homing MMPs exhibit specific molecular characteristics that enable them to settle in the thymus: 1) To migrate and enter the thymus they express CCR7 and CCR9 (55, 56), and 2) they should be responsive to NOTCH signaling (54), ultimately these thymus-homing MMPs are known as thymus seeding progenitors (TSPs). TSPs are a heterogeneous group of multipotent, non-committed progenitors that selectively home to the thymus (57). Multiple candidates have been proposed as TSPs, including multipotent progenitors (MPPs), lympho-myeloid primed progenitors (LMPPs), common-lymphoid progenitors (CLPs) or T cell-lineage committed progenitors (58). Identifying TSPs is challenging due to rapid changes in cell-surface phenotypes, transcripts, and the extremely limited numbers of these progenitors in circulation and the adult thymus (58–60). Further research is required to establish a consensus on the various subsets constituting the group of TSPs, as well as to develop a unified nomenclature for clarity in the field (see **Table 1**).

**Table 1:** Compilation of the different progenitors subsets identified as TSPs candidates in the bone marrow or blood and the progenitors subsets identified in the thymus with their corresponding flow cytometric profile.

Tissue	Subset name	Abbrev.	Gating strategy/profile	Ref.
BM	Hematopoietic Stem Cells	HSCs	Lin^-^ Sca-1^+^ c-Kit^hi^ Flt3^-^CD34^+/-^ [m] [FC] Lin^-^ Sca-1^+^ c-Kit^hi^ Flt3^-^CD34^+/-^ CD48^-^ CD150^+^ [m] [FC]	(61–63)
Thymus and BM	Lineage^-^ Sca-1^+^ c-Kit^+^	LSK	Lin^-^ Sca-1^+^ c-Kit^+^ [m] [FC]	(55, 60)
Blood	Circulating T cell progenitor	CTPs	Lin^-^ Sca-1^+^c-Kit^lo^ CD25^-^ CD44^+^ Thy1.1^+^ IL-7Rα^+^ PSGL-1^+^ CCR9^+^ [m] [FC]	(64)
Thymus and BM	Lympho-Myeloid Primed Progenitors	LMPPs	Lin^-^ Sca1^+^ c-Kit^+^ Flt3^hi^ [m] [FC] Lin^-^ Sca1^+^ c-Kit^+^ CD34^+^ Flk2^+^ [m] [FC] Lin^-^ Sca1^+^ c-Kit^+^ CD34^+^ IL-7Rα^-/lo^	(36, 65, 66)
Bone marrow	Common-Myeloid Progenitors	CMPs	Lin^-^ c-Kit^+^ Sca-1^-^ CD34^+^ CD16/32^lo^	(67)
Thymus and BM	Common Lymphoid Progenitors	CLPs	Lin^-^ c-Kit^+^ IL-7-Rα^+^ [m] [FC] Lin^-^ Sca-1^lo^ c-Kit^lo^ Flt3^hi^ IL-7-Rα^+^ [m] [FC] Lin^-^ Sca-1^+^ c-Kit^+^ IL-7-Rα^+^ CD34^lo/-^[m] [FC]	(36, 60, 65)
BM	Common Lymphoid Progenitors-2	CLP-2	Lin- c-Kit^-/lo^ B220^+^ [m] [FC][cc]	(60)
BM and Thymus	Granulocyte-monocyte Progenitors	GMPs	Lin^-^ CD4^-^ CD34^+^ CD1a^-^ CD44^+^ HLA-DR^+^ CD123^+^ IRF8^hi^ [h] [CS] Lin^-^ Sca1^-^ c-Kit^+^ CD34^+^ CD16/32^+^ [m] [FC]	(36, 48, 68)
BM	Early Lymphoid Progenitors	ELPs	Lin^-^ c-Kit^hi^ Sca-1^hi^ CD27^+^[m] [FC]	(69)
Thymus	Early Thymus Progenitors	ETPs	Lin^-^ CD25^-^ CD44^+^ c-Kit^hi^ [m] [FC] Lin^-^ CD25^-^ CD44^+^c-Kit^hi^ IL-7Rα^+^ Sca-1^+^ [m] [FC]	(31, 55, 70)
Thymus and BM	Macrophage - Dendritic progenitors	MDPs	Lin^-^ c-Kit^hi^ Flt3^+^ CX3CR1^+^	(71)
Thymus and BM	Common Dendritic Progenitors	CDPs	Lin^-^ c-Kit^hi^ Flt3^+^ CX3CR1^+^ CD115^+^	(71)

^-/lo^, negative/low expression; ^lo^, low expression; ^+^, positive expression; ^hi^, high expression; BM, Bone marrow. Analogous names for antibodies: c-Kit, CD117; Sca-1, Ly-6A/E and Ly-5.1; Flt3, CD135 and Flk2; CD150, SLAMF1; CD48, SLAMF2; IL-7Rα, CD127; CD16/32, FcγRIII/FcγRII; CD115, CSFR1; CD123, IL-3Rα; Thy1.1, CD90.1; CCR9, CD199. Symbols: [m], mouse; [h], human, [FC], Flow cytometry; [CS], CITE-Seq; [cc], cell culture, in vitro observation.

TSPs consist of lympho-myeloid primed progenitors (LMPPs) (57, 72), common lymphoid progenitors (CLPs) (65, 73), and granulocyte-monocyte progenitors (GMPs) (36, 48, 68). Shortly after entry, CCR9 is downregulated by NOTCH signaling (74). The environmental cues by the thymus stroma influence TSPs, leading them to adopt the phenotype of early thymus progenitors (ETPs) (54). Murine ETPs have a great proliferative potential (75), with a single progenitor being able to give rise to more than 10^5^ thymocytes in 12 days of fetal thymic organ cultures (FTOCs), implying a doubling time of 34 h on average (76). The doubling time of human ETPs has not been well characterized, likely due to technical limitations (59, 77). Constitutive expression of *Notch1* in bone marrow progenitors leads to loss of B cell potential and aberrant T cell development (49). The presence of NK/T-biased (B-cell deficient) progenitors in the fetal liver and fetal blood suggests that NOTCH signaling might occur extrathymically prior thymus colonization (49). More recently, it has been shown that bone marrow LMPPs undergo NOTCH signaling resulting in inhibition their myeloid potential and skewing toward T cell fate (72). Furthermore, DN1a/b cells from mice RBPJ-inducible mice (unresponsive to NOTCH signaling) exhibited a myeloid bias (72). These findings demonstrate that NOTCH signaling pre-primes TSP progenitors extrathymically and is required before thymus colonization (72).

Throughout development, particularly in fetal and neonatal stages, there are different reservoirs of HSCs. The yolk-sac (YS) is the earliest niche of HSCs, exporting progenitors from E7.5 (78, 79). Subsequently, the aorta–gonad–mesonephros (AGM) contributes to the circulating HSCs pool from day E9. Progenitors from the YS and AGM seed the fetal liver that actively export HSCs to peripheral organs from E10.5, a process that gradually decreases during development (78, 79). Eventually, the bone marrow becomes the primary HSC reservoir from E14.5 onwards, dominating HSC export by E17 (78, 79) (**Figure 2**). The earliest stage of fetal thymus colonization in mice is detected at day E11.5 (29). Progenitor cells migrating from various hematopoietic niches to the thymus are guided by G protein-coupled receptors (GPCRs), responding to chemokine gradients towards the thymus (80). The first step of mobilization of hematopoietic progenitors from the bone marrow into circulation is regulated by the CXCR4-CXCL12 axis (54). *Cxcr4* ^-/-^ mice exhibited impaired migration from the bone marrow to the fetal liver (81). Additionally, c-Kit - stem cell factor (SCF) axis was also identified being involved in the mobilization of bone marrow progenitors into circulation (54). The fetal thymus produces CCL21, CCL25, and CXCL12, chemoattractants for TSPs (54). Blockage of CCL21 and CCL25 but not CXCL12 leads to reduced colonization of TSPs progenitors in the thymus (79). The number of fetal thymocytes is reduced in CCR7 knock-outs (receptor for CCL21) (79) and CCR9 knock-outs (receptor for CCL25) (82), while CXCR4 (receptor for CXCL12) knock-out mice were not defective in fetal thymus colonization. Various studies employing CCR7/CCR9 double knock-outs independently demonstrated that CCR7-CCL21 and CCR9-CCL25 are essential in regulating thymus homing of uncommitted progenitors towards the thymus prior and after vascularization (29, 56).

### 3.2 T-cell development and thymic selection in space and time

**Figure 3:**
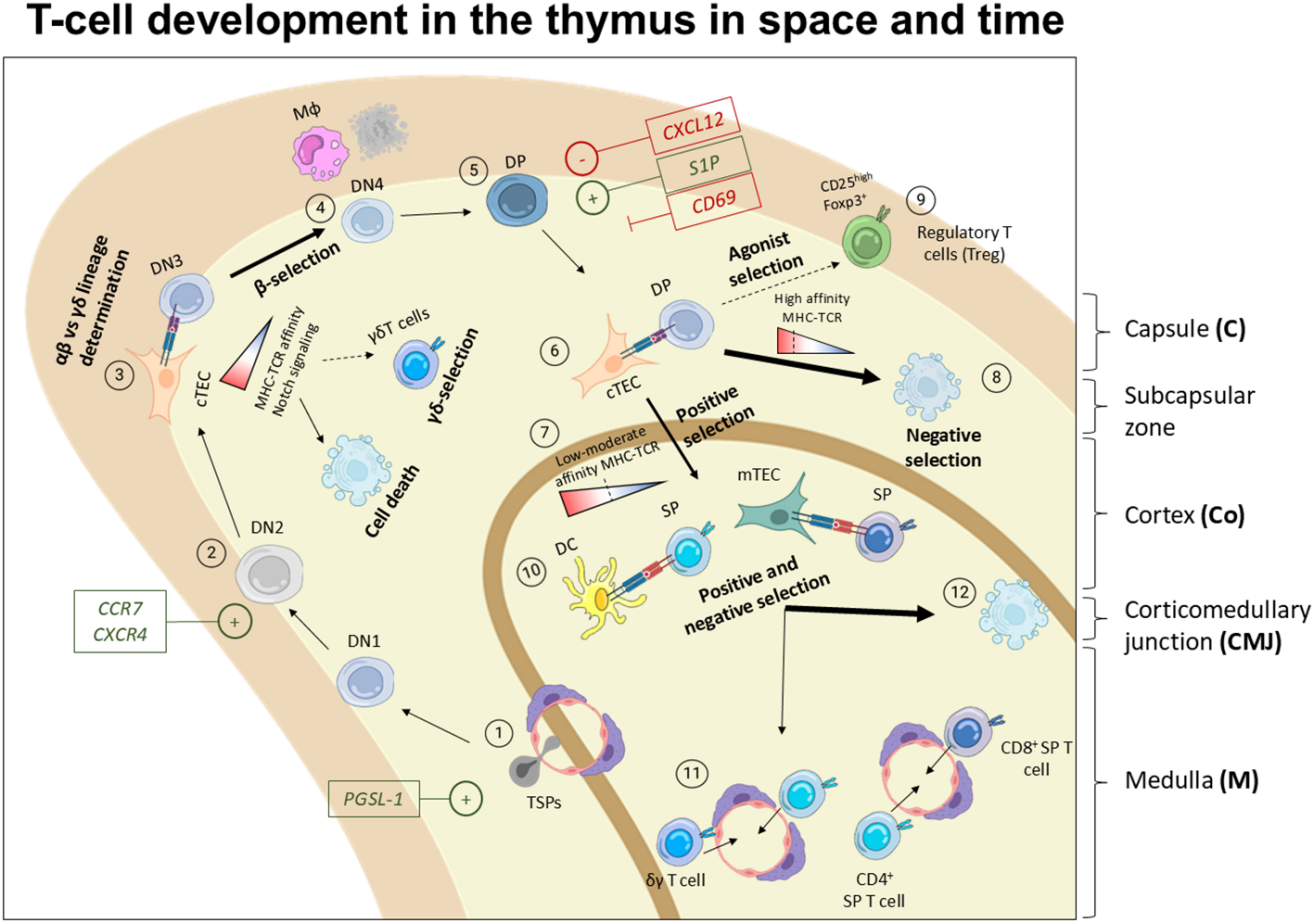
Spatiotemporal model of T cell development in the thymus. 1) TSPs enter the thymus through blood vessels typically irrigating the thymus in the medulla and CMJ in a process mediated by PGSL-1. Then, the thymus microenvironment instructs them to become ETPs or DN1 entering the T cell differentiation path. 2) DN1 move towards the cortex where they become DN2 which proliferate robustly. This migration through the cortex is mediated by chemokine gradients promoting the migration of the developing thymocytes via CCR7 and CXCR4. 3) In the outer cortex, DN3 undergo TCR rearrangement, thereby irreversibly committing towards T cell fate. 4) Next, DN thymocytes encounter αβ- vs γδ- lineage choice based on the MHC-TCR interaction strength, and NOTCH signaling among other factors. In this step, their TCR is tested in a process known as β- or γδ- selection, where cells must interact with MHC to receive a survival signal. Failure to engage MHC results in programmed cell death at the DN3 stage. 5) Later on, DN3 move forward in the development and become DN4 and DP, staying quiescent for several days. At this point, the polarization of the migration reverses. This inward migration of DP is regulated by S1P, CXCL12 and CD69. 6) DP interact with cTECs and undergo positive selection based on the TCR-MHC strength and affinity interactions. 7) Cells displaying low-moderate TCR-MHC interaction are positively selected. 8) Cells with high TCR-MHC affinity mostly undergo cell death, 9) while a small fraction that recognize self-antigens are rescue to become regulatory T cells (Tregs) or other types of unconventional T-cells (95). 10) Positively selected cells undergo another selection round by interacting with APCs, either DCs or mTECs. 11) Successful interactions lead to their maturation into SP CD4^+^ or CD8^+^ thymocytes and naïve T cells that are ready to egress to the periphery and complete their development in secondary lymphoid organs.

TSP progenitors enter the thymus at the CMJ region from the blood by transmigration and extravasation (46), a process regulated by P-selectin glycoprotein ligand-1 (PSGL-1), CCR7, and CCR9 (83) (**Figure 2**). Once in the thymus, most of the TSPs are instructed by the thymus microenvironment becoming ETPs and mostly being directed towards T cell fate. However, ETPs comprise a heterogeneous group of non-committed progenitors that can adopt granulocyte- (36, 84), monocyte/macrophage- (45, 85), dendritic- (86, 87), ILCs- (31, 73), B- (88) and, NK- (35, 89) fates. Following initial contact with thymus milieu and the thymic stroma, the TSPs become early thymic progenitors (ETPs), and remain uncommitted towards T cell fate (46, 53). Downstream ETPs, double negative (DN) thymocytes, characterized by the lack of CD4 and CD8 surface markers, comprise the different maturation stages of developing T cells. DN cells undergo a stepwise differentiation pathway orchestrated by the thymic stroma and supportive immune cells that consist in two main phases; specification and commitment (51). DN1 (Lin^-^ CD44^+^ CD25^-^) are located close to the entry site in the corticomedullary junction. DN1 survival is promoted by IL-7 - IL7R and stem cell factor (SCF) - c-KIT interactions (90, 91), while Delta-like ligand 1 (DLL1) – NOTCH1 axis promotes differentiation (92), and CCR7 aid their progressive migration toward the cortex and subcapsular zone (93, 94). As they move through the medulla, they transition into the DN2 stage (Lin^-^CD44^+^CD25^+^). CCR7 deficient mice accumulate DN1-2 cells in the corticomedullary junction, unveiling that CCR7 is essential for controlling the migration of DN1-2 cells from the corticomedullary junction to the cortex (93). Additionally, CXCR4 deficient mice show an arrest in T cell development at DN1 stage, revealing the importance of CXCR4 in facilitating the migration of DN1 cells to the cortex (93) (**Figure 3**). Despite the roles of CCR7, CCR9, CXCR4, our understanding of the environmental cues that direct the migration of early DN thymocytes from the CMJ to the cortex remains uncomplete (96). DN1 thymocytes are multipotent, while DN2 thymocytes are uncommitted to T cell lineage but their fate is restricted to B-, T-, NK-, and DCs- lineages (94).

The commitment towards T cell fate starts at DN2a stage by upregulation of IL-7R, and components of the TCR complex (97). At DN2b stage, the progenitors experience an upregulation of transcription factors and genes linked to the rearrangement of the TCR, leading to irreversible commitment to T cell fate (92, 97, 98). DN2 thymocytes proliferate extensively before TCR rearrangement (99). Mice deficient for RAG1 or RAG2 recombinases show a complete block in T cell development at DN3 stage (100). In the subcapsular zone, where DN3 cells reside, part of the subset undergoes β-selection (99) where the strength of the TCRβ chain is tested in combination with a surrogate “pre-TCR” before the TCRα chain is created (101). With no lysine kinase 1 (WNK1) is serine/threonine-specific kinase is expressed in developing T cells that can regulate the activity of solute carrier transporters (SLCs) ultimately promoting cell motility. Notably, both TCR- and CCR7- signaling results in WNK1 upregulation that negatively controls LFA-1-mediated ICAM adhesion, promoting chemokine-mediated cortical migration of β-selected DN3 thymocytes (102). At the same time, fewer DN3 cells undergo γδ-selection where the newly form γ- and δ- chains are functionally tested (51).

Notably, γδ T cells constitute less than 0.5% of the total murine thymocyte population (103). DN cells expressing TCRγδ commit to the γδ-lineage, typically without entering the DP stage of T cell development (104). Whereas most γδ T cells remain DN and become mature γδ T cells prior to egressing the thymus, some acquire CD4 and CD8 markers suggesting that they undergo a similar DP-to-SP development route as αβ T cells (104). The αβ vs γδ lineage choice is currently debated between by two different models. Firstly, the classical model includes a pre-commitment selection where the lineage fate is determined before TCR-rearrangement. Secondly, the alternative model emphasizes the TCR signal strength and NOTCH signaling, rather than TCR identity, to dictate αβ vs γδ lineage fate, with strong TCR signal favoring γδ-skewing (103, 104). DN3a thymocytes, characterized by low expression of CD27 can differentiate into TCRγδ T cells in the absence of NOTCH/Delta signaling while the absence of NOTCH stimulation in DN3a cells reduce the number of TCR-αβ-lineage thymocytes (105). In mice, the NOTCH ligand JAG2, promotes γδ T cell development, while DLL1 and DLL4 contribute to αβ lineage development (104). OP9-DL1 cultured human *Il2* ^GFP^ DN3b thymocytes preferentially adopt γδ lineage over αβ (105). This subset was characterized as CD27^+^ CD5^+^ CD2^+^ CD62L^+^ in accordance with the expression pattern of differentiated γδ T cells (105). This study revealed differential gene expression of *Runx3*, *Egr-2*, *Egr-3*, *Id3*, *Ikaros*, *Bcl-2*, *Helios* and *Aiolos* in murine γδ T cells compared to other thymocyte subsets. Other studies showed increased *NR4A1-3*, *ETV5*, *KLF2*, *RELB*, *HES1*, and *ZBTB16* in human γδ T cells (106, 107). Nonetheless, the identity of the supposed γδ-precursor within the DN fraction is still unknown. In mice, a DN1 subset expressing high levels of IL-7 and SOX13 preferentially adopt γδ fate (108, 109). Collectively, the two prevailing developmental models are not opposed to each other as both pre-TCR progenitor identity, TCR-signal strength, and NOTCH signaling seem to contribute to γδ-lineage development (110).

While γδ-selected DN3 thymocytes show little proliferation and remain double negative, DN3 thymocytes that underwent β-selection proliferate robustly and rapidly differentiate into DN4 cells (Lin^-^CD44^-^CD25^-^) and later acquire CD4 and CD8 surface markers becoming double-positive (DP) thymocytes (CD44^-^CD25^-^CD4^+^CD8^+^) in the subcapsular zone (51) (**Figure 3**). DP thymocytes remains quiescent for several days as the TCRα rearrangement process involves the creation of double-strand breaks in the DNA being incompatible with DNA replication (111, 112). At this stage, the polarity of the migration of DP T cells reverses, guiding them back from the cortex to the medulla (113). Gradients of CXCL12 (SDF-1) repel thymocytes, while S1P_1_ acts as a chemoattractant, aiding their migration across the thymus layers (113). CD69, which antagonizes S1P_1_, serves as a residency marker (113). In the cortex, DP T cells interact with cTEC, DCs, and B cells as antigen presenting cells (APCs) (114, 115). A weak TCR:MHC interaction is required to protect DP thymocytes from death by neglect, known as positive selection (116–118). Positively selected CD4^+^ or CD8^+^ SP thymocytes are relocated into the medulla. There, they engage with DCs, mTECs, and plasmacytoid dendritic cells (pDCS) for further screening. SP Thymocytes that show too strong TCR: MHC-self peptide interactions undergo negative selection and die predominantly by apoptosis (91, 115). Notably, SP thymocytes exhibiting high-affinity TCR:MHC-self peptide interactions, considered autoreactive, can be redirected to become regulatory T cells (Treg), invariant natural killer T cells (iNKT) or TCRαβ^+^ CD8αα^+^ intestinal intraepithelial lymphocytes (IELs), in a process known as agonist selection (91, 119).

A small fraction of thymic SP CD4^+^ T cells (∼1%) expressing the high-affinity IL-2 receptor alpha (IL-2Rα) or CD25 and displaying strong affinity for self-peptides, as revealed in *Nur77* ^GFP^ transgenic reporter mice, are agonist-selected to become Tregs (120, 121). Initial TCR stimulation causes upregulation of CD25, GITR, OX40 and TNFR2 among others (121). CD4^+^ CD25^hi^ thymic Treg precursors subsequently acquire FOXP3 expression independently of further TCR engagement in the presence of IL-2 or IL-15 (122). FOXP3 expression is tightly regulated at the transcriptional level by four conserved non-coding sequences (CNS1-4) (95). Transcription factors bind these CNSs to control *Foxp3* transcription. Notably, c-Rel binding to CNS3 is crucial for thymic Treg development, as evidenced by the dramatic decrease of thymic Tregs in *c-Rel^-/-^* mice (103). Interestingly, continuous endogenous production of IFN I and IFN III by AIRE^+^ mTECs is required to instruct APCs to promote Treg selection (42). While the thymus is the major site for Treg development, around ∼10–15% of peripheral CD4^+^ T cells can differentiate into Tregs instructed by environmental cues such as the presence of TGFβ (121, 123). Much less is known about the signals directing intrathymic Treg precursor migration, and the residency or egress of Tregs compared to their conventional T cell counterparts. Notably, the maintenance of immunological self-tolerance highly relies on Treg suppressive functions as unveiled by the lethality of *Foxp3^-/-^* murine model (124, 125) and the *Foxp3* mutation in humas leading to immune dysregulation, polyendocrinopathy, enteropathy and X-linked (IPEX) syndrome ultimately resulting in death during infancy (126).

Ultimately, successfully selected naïve SP CD4^+^ and CD8^+^ T cells, CD4^+^ CD25^+^ FOXP3^+^ Tregs, and other unconventional T cells subsets egress the thymus via lymph and blood vessels and migrate to secondary lymphoid organs such as lymph node where they complete their maturation and exert their functions.

### 3.3 Hormone regulation of intra-thymic T-cell migration and survival

Beyond its crucial role as a primary lymphoid organ and its contribution to the establishment of the lifetime T cell pool, the thymus also functions as an endocrine gland (127, 128). Accordingly, the thymus is highly innervated by both sympathetic and parasympathetic nerves, facilitating neuroendocrine control and similar to the adenohypophysis, thyroid, adrenals releases hormones into the bloodstream (127, 129). Neurotransmitters such as norepinephrine (NE) and acetylcholine (ACh) are locally released and control the delivery and expression of other hormones (128).

The thymus produces several hormones like thymosin, thymopoietin, thymulin, thymic humoral factor (THF) and luteinizing and follicle-stimulating hormones, which are important in pre-thymocyte maturation (127, 129–132). Hormones play a critical role in influencing cell motility and contributing in the regulation of life versus death decisions of developing T cells. Thymulin is an important regulator of neuro-immune endocrine thymus axis (130). Paracrine signaling via thymopoietin specifically enhances the differentiation of T cells (131), and thymosin is essential for pre-thymocyte maturation (127, 129). Outside the thymus, thymosin, thymopoietin, and thymulin seem to be highly immunomodulatory but are also part of an extensive neuroendocrine network, affecting the production of other hormones, such as the production of pineal gland hormones affecting the circadian rhythm (133). Thymopoietin have been shown to directly bind to antigen-presenting cells on their MCH class II molecules, whereas thymulin and thymosin seem to retain anti-inflammatory functionalities (134–136).

Stress- and Age- related hormonal changes are known to influence thymic output (137–139). Endocrine hormone signaling significantly influences thymocyte development and output by regulating cell migration controlling the degradation or deposition of the thymus extracellular matrix components (ECM) thereby affecting chemokine-ECM intrathymic migration routes (137). For example, growth hormone (GH) and prolactin can boost thymocyte growth and movement (140, 141). Additionally, prolactin affects DCs-expressing prolactin receptors resulting in the increase production of proinflammatory cytokines leading to acute thymus atrophy induction (141). Thyroid hormones like triiodothyronine (T3) and thyroxine (T4) also play a role in T-cell development by affecting thymocyte adhesion and migration (137). Notably, glucocorticoids are known to trigger apoptosis in developing T cells (142), whereas leptin, primarily produced by adipose tissue crucially impact mTECs-expressing leptin receptor conferring protection against thymic stress and enhancing thymus output (143). Oxytocin (OT) and vasopressin (VP), mainly produced in the pituitary gland, can also be produced in the thymus and impact SP CD8^+^ thymocytes, leading to apoptosis and reduced proliferation (144).

In mammals, sex hormones like androgens and estrogens are hypothesized to contribute to thymic decline with age, as evidenced by the decline of thymus size and hormonal changes post-puberty (128). In line with this hypothesis, sex steroid hormones ablation in mice leads to increase expression of Delta-like 4 (Dll4) in TECs, ultimately improving thymic function (145). In rats, maternal protein deprivation during lactation resulted in increased endogenous leptin levels protecting thymocytes from apoptosis (143). Conversely, ghrelin enhances thymopoiesis in aged mice, and its diminished expression in the thymus with age suggests a protective role against age-related involution by increasing ETPs numbers in aged mice (139, 146).

Altogether, developing T cells migration, differentiation, maturation, proliferation, and survival are tightly regulated by a variety of hormones produced both locally and in other endocrine glands, critically impacting thymus homeostasis and output.

### 3.4 Regulation of thymocyte migration and survival by the thymus extracellular matrix

The thymus extracellular matrix (ECM) is a complex network of proteins, glycosaminoglycans, and other molecules that support and facilitates T cell development in combination with other thymus immune-, and stromal cells (147). Thymic stromal cells, including epithelial cells and fibroblasts, actively produce ECM components such as collagen. The main ECM components include: collagen I, collagen III (to a lesser extent), collagen IV, fibronectin, and laminins (148–150). Collagen I is predominantly found in the intracapsular and intraseptal fibers, while collagen IV, fibronectin, and laminin are defining the membranes in the capsula, septa, and perivascular spaces (151). These molecules are known to mediate adhesion, migration, and differentiation of thymocytes (152). Thymic myeloid cells express various metalloproteases (MMPs) that degrade collagen fibers, influencing the intrathymic migration routes, which are essential for interactions with thymic epithelial cells and developmental progression of T cells (153–155).

Thymocytes move through the inner thymus in an integrin-dependent manner with ECM components modulating their adhesion and spatially regulating thymocyte development. *In vitro* experiments showed that thymocyte-fibronectin specific interactions by recognizing the amino acid sequence Gly-Arg-Gly-Glu-Ser-Pro regulate the migration of developing thymocytes in a gradient-dependent manner (156). Developing thymocytes also engage the ECM via CD44, that binds hyaluronic acid and collagen (151), and adhere to laminin via α6β64 integrin. Additionally, VLA-4, -5, -6, and LFA-1 are important regulators of the rolling and adhesion motility of thymocytes to the ECM (151, 157). Laminins, primarily produced by thymic stromal cells form gradients and allow integrin-laminin specific interactions that create intrathymic routes for thymocyte development (158).

Notably, hormones also orchestrate ECM remodeling thereby controlling developing thymocytes migration and specification. Laminin production and secretion by TECs is regulated by growth hormone (GH) (158). Similarly, triiodothyronine (T3) treatment increased laminins and VLA-6, leading to an enhanced migration of thymocytes (158). Glucocorticoids (GCs) are steroid hormones that cross the plasma membrane and bind to an intracellular glucocorticoid receptor. Produced by thymic epithelial cells (TECs), GCs shapes thymocytes TCR repertoire by mitigating downstream TCR signaling events, thereby promoting thymocyte negative selection. For example, GC treatment independently inhibits the transcription factors *Nur77* and *Helios*, which are upregulated in TCR-signaled thymocytes (159). Dexamethasone (Dex), a synthetic glucocorticoid commonly used in clinical settings, induces extensive thymocyte apoptosis, resulting in acute thymus atrophy. Dex administration triggers apoptosis through phosphoinositide-specific phospholipase C (PLC) and acidic sphingomyelinase (aSMase) signaling, leading to subsequent caspase-3 activation (160). Additionally, Dex treatment reduces NFAT, AP-1, and c-MYC activity in thymocytes and limits the expression of anti-apoptotic factors like BCL-2 by promoting proteasomal degradation, ultimately causing acute thymus atrophy (161). Additionally, GCs can stimulate the TECs leading to the accumulation of laminins between the cortex and medulla, therefore impairing DN4-DP migration leading to T cell development arrest and thymus atrophy (158, 162).

Taken together, the secretion, distribution, and breaking down of the ECM components are tightly regulated by thymus immune- and stromal subsets together with hormones, playing an active role in remodeling the thymus landscape, thereby promoting or limiting the developmental progression of pre-T cells.

### 3.5 Non-T cell thymic subsets contribution to thymus homeostasis and T cell development

Beyond developing T cells, the thymic immune landscape also contain other immune cell substes including B cells (114, 163), DCs (48, 68), eosinophils (32), macrophages (34), neutrophils (36, 164), and all three groups of ILCs (31, 165–168), each of them performing an essential role in maintaining thymus homeostasis and enabling T cell development. Thymic B cells participate in negative selection and tolerance acquisition by presenting autoantigens and contributing to the development of regulatory T cells (114, 163). Thymic DCs, together with thymic epithelial cells (TECs) engage in MHC-TCR interactions with developing T cells and present self-antigen playing a crucial role in the progression or clonal deletion of developing T cells (169, 170). Macrophages, neutrophils, and eosinophils act as scavengers eliminating the dying T cells in a very rapid process (117, 164, 171). Additionally, thymus macrophages functions extends beyond phagocytosis, as they can present antigen and induce cell death of self-reactive thymocytes (172). Although the functions of thymic ILCs during homeostasis remain largely unexplored, some studies suggest that ILCs can influence fate decisions of uncommitted thymus progenitors, and impact thymus epithelial cells that control the maturation and egress of developing T cells (165, 166).

### 3.6 Selection checkpoints: life and death of a developing T-cell

T cell development and selection is highly regulated to avoid self-reactivity and autoimmune disorders. During its most productive phase, the mouse thymus generates around 50 million of DP thymocytes each day that undergo a series of selection processes. In total, it is estimated that only 3-5% of developing thymocytes become mature CD4 or CD8 single positive (SP) T cells and exit the thymus (117). Programmed cell death (PCD) plays a crucial role in the elimination of a large number of Pre-T cells that do not overcome either β-, δ-, negative, positive or agonist selections to prevent autoimmunity (59). The specific molecular cell death pathways downstream the different T-cell development checkpoints have not been fully mapped (112, 173), although apoptosis is the default death modality during T cell development (174, 175).

Early during T cell development, DN1-2 survival is controlled by the expression of the cytokine receptors CD117 (c-KIT) and IL-7R that triggers anti-apoptotic signaling via Bcl-2 and the availability of their respective ligands SCF and IL-7 (176). After the DN2 stage, thymocyte survival depends on the correct expression of the TCRβ chain and the surrogate pre-TCRα chain in a process known as β-selection. BCL-2 is expressed both in DN and SP thymocytes, whereas BCL-XL is predominantly expressed in DP cells (177). Thymocytes that fail to bind self-MHC die by caspase-3 dependent apoptosis (178). NF-kB dependent activation of BCL-2-related protein A1 (BCL2A1) is required to inhibit caspase-3 dependent of DN thymocytes (178). At this stage, CXCR4 is required for the progression through β-selection as regulator of the localization of DN1-2 thymocytes and as a co-stimulator to promote the pre-TCR dependent activation of BCL-2 (179). Additionally, FAS-associated protein with death domain (FADD) plays a critical role in regulating survival and transition of DN2 and DN3 during β-selection by modulating NOTCH signaling (180).

The BCL-2 family member BIM is required for apoptosis of autoreactive SP CD4^+^ and CD8^+^ thymocytes. Indeed TCR ligation upregulated Bim expression and promoted interaction of BIM with BCL-XL, inhibiting its survival function (181). This demonstrates the engagement of the intrinsic mitochondrial pathway during negative selection. *Bim* ^-/-^ mice showed abnormal T cell development characterized by loss of DP T cells and accumulation of DN an SP subsets (182). *In vivo* deletion of *Bid* and *Bim* suppressed pre-TCR thymocyte cell death (183). DP thymocytes with appropriate TCR:MHC interaction strength are positively selected, becoming naïve SP CD4^+^ or SP CD8^+^ T cells (184). However, the majority of DP thymocytes (approximately 90%) are unresponsive to TCR:MHC engagement (184). These cells become sensitized to BIM-mediated intrinsic apoptosis due to BCL-2 downregulation, and ultimately undergo death by neglect (185). The MAPK pathway is upregulated in DP thymocytes that exhibit low TCR:MHC avidity, leading to their positive selection (186) while “strong” negatively selecting signals lead to Bim-mediated intrinsic apoptosis (181, 187).

Notably, deletion of *Mcl-1* results in thymocyte cell death, a phenomenon rescued by additional deletion of *Bak*, but not by single deletion of *Bak* or *Bim* (188). This suggests that MCL-1 promotes thymocyte survival independently of BCL-2 by sequestering proapoptotic Bak (188). Interestingly, thymocytes from *Bax* ^-/-^ *Bak* ^-/-^ double-knock out mice were resistant to death-by-neglect (189). However, these mice displayed reduced thymopoiesis over time, suggesting that the elimination of apoptotic cells is needed for restoration of normal T cell development. Noteworthy, *Bid* ^-/-^ *Bim* ^-/-^ *Puma* ^-/-^ triple-knockout mice displayed a large thymus, and T cells were resistant to IL-7 deprivation (190). Mechanistically, the triple deletion of *Bid*, *Bim*, and *Puma* prevented the oligomerization of BAX and BAK, and subsequent cytochrome c–mediated release of caspases, thereby blocking intrinsic apoptosis (190).

**Figure 4:**
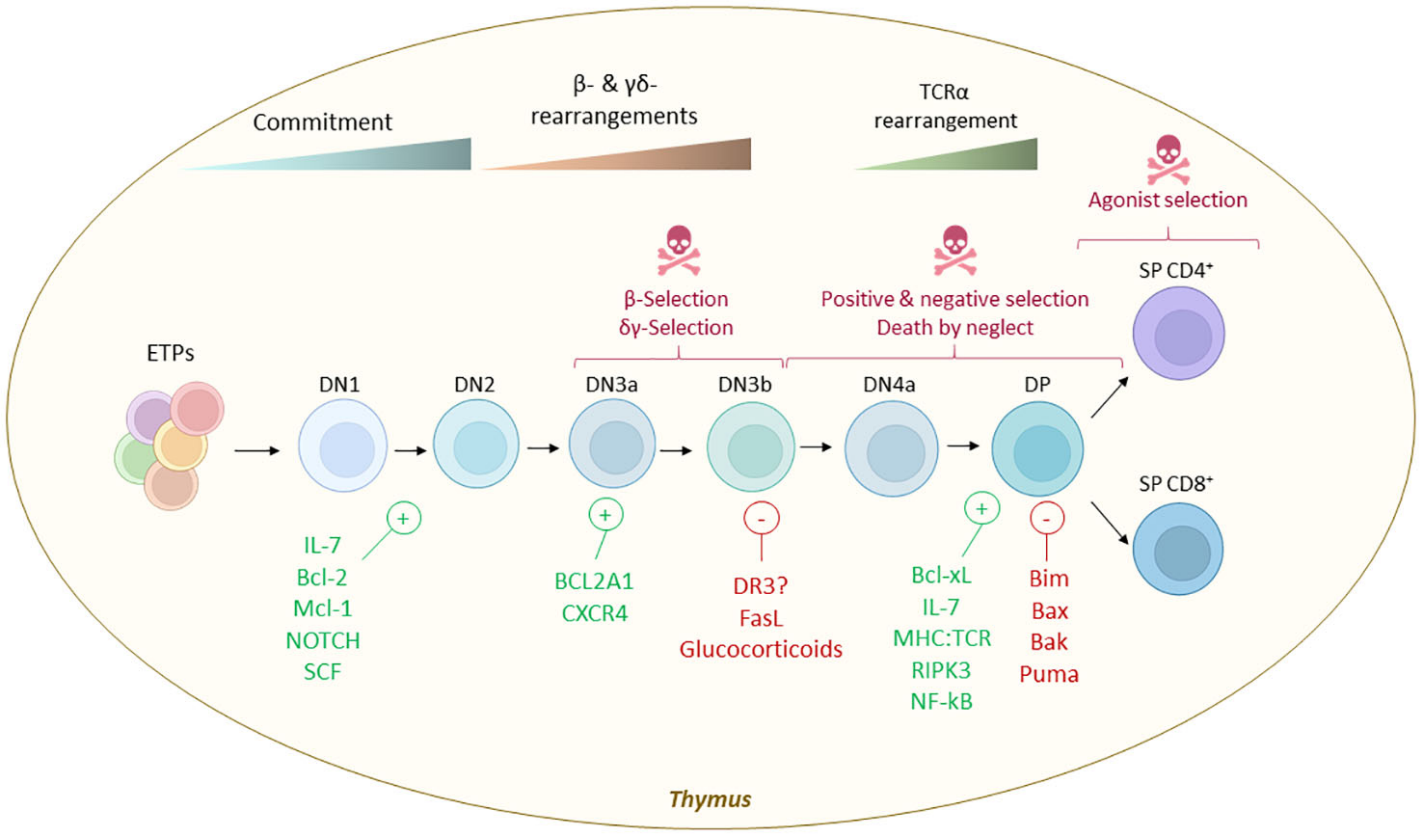
Cell death selection checkpoints during thymic T-cell development and molecules that control life vs death decisions. Early thymus progenitors (ETPs) are uncommited progenitors that enter the T cell differentiation path instructed by the thymus stroma. The earlier stage is known as DN1 (defined as Lin^-^ CD44^+^ CD25^-^ c-Kit^hi^), later becoming DN2 (defined as Lin^-^ CD44^+^ CD25^+^ c-Kit^+^). The survival of these progenitors is controlled mainly by IL-7 availability, SCF - c-KIT interactions, NOTCH signaling and BCL-2 and MCL-1. Later, when the machinery of TCR recombination these cells are irreversibly committed to the T cell fate and become DN3a (defined as Lin- CD44^-^ CD25^+^ c-Kit^-/lo^ CD27^+^ CD28^-^) and later DN3b (defined as Lin- CD44^-^ CD25^+^ c-Kit^-^ CD27^+^ CD28^+^). During this stage the TCR chain is tested and BCL2A1 and CXCR4 were reported to control the survival of DN3 while FASL and glucocorticoids promote cell death when the TCR is defective. Then, DN3 cells move forward in the development acquiring a DN4 phenotype (characterized as Lin- CD44^-^ CD25^-^ CD27^+^ CD28^+^) and ultimately a DP phenotype (characterized as CD4^+^ CD8^+^). These cells remain quiescent for some days and later undergo additional selection rounds where the TCR:MHC affinity is tested. TCR:MHC interaction, BCL-XL, IL-7, RIPK3 and NF-κB are some of the signals that dictates their survival, while BH3-only proteins BIM and PUMA and their mitochondrial pore forming targets BAX and BAK are implicated in the cell death execution when the TCR:MHC interaction is not successful. Lastly, DP thymocytes will adopt a SP CD4^+^ or CD8^+^ phenotype and will egress from the thymus to secondary lymphoid organs where they complete their maturation into different effector and helper subsets. A small fraction of SP CD4+ cells that show high TCR:MHC interaction can be rescued from cell death to become regulatory T cells (T_regs_, defined as CD4^+^ FOXP3^+^ CD25^+^) or unconventional T cells (iNKT, MAIT, etc.).

Notably, many key contributors to the survival and developmental progression have been identified (**Figure 4**). While most studies emphasize the involvement of intrinsic apoptosis as the primary cell death mechanism through T cell selection, the death receptors tumor necrosis factor receptor 1 (TNFR1), death receptor 3 (DR3), and death receptor 5 (DR5) are expressed in DN3-4 thymocytes suggesting a role in thymic selection (191). Interestingly, RIPK1 expression is developmentally controlled and it is only expressed following positive selection (192). Deletion of *Ikk1* and *Ikk2* with the hCD2^iCre^ (DN2 cells and downstream) or CD4^Cre^ (DP cells and downstream) resulted in reduced numbers of SP CD4^+^ and CD8^+^ that were more sensitive to TNF-induced extrinsic apoptosis, while DP and DN numbers remain unaffected (192, 193). TNF-mediated NF-κB pathway and IKKs are key in controlling RIPK-kinase activity and promoting SP survival (192). IKKs control SP survival by repressing RIPK1-induced cell death independently of NF-κB signaling. Noteworthy, despite sensitizing CD4^+^ or CD8^+^ SP thymocytes to TNF-induced extrinsic apoptosis, deletion of RIPK1 in thymocytes, does not result in reduced numbers or frequencies of DN, DP, or CD4^+^ or CD8^+^ SP thymocytes *in vivo* (193). These results suggest that RIPK1 and RIPK1-kinase activity are not essential for the steady-state survival and developmental progression of thymocytes. Additional deletion of caspase-8 (*Cd4* ^Cre^ *Ripk1* ^-/-^ *Casp8* ^-/-^) did not affect thymus development excluding engagement of RIPK1-mediated mechanisms (193). However, caspase-8 has been proposed to play a role in thymocyte development and elimination (194–196). Indeed, while caspase-8 activation in DP thymocytes was shown to be FAS-independent, the medullary CD4^+^ or CD8^+^ SP thymocytes that exhibit strong TCR:MHC interaction underwent FAS-dependent Caspase-8 mediated extrinsic apoptosis in a model of staphylococcal enterotoxin B (SEB), resulting in clonal elimination of SEB-reactive Vβ8^+^ cells (197).

Besides extrinsic apoptosis by death domain receptors, also the involvement of other cell death mechanisms such as RIPK3/MLKL-driven necroptosis in thymocyte development have been investigated. Thymus morphology and macrostructure remained largely unchanged in necroptosis deficient *Mlkl* ^-/-^ and *Ripk3* ^-/-^ mice compared to littermate controls (198). Further characterization of the RIPK3-deficient mice revealed abnormal proliferation of DP thymocytes, leading to thymus hyperplasia and an increased incidence of thymomas (199). This study suggests that RIPK3 may play a role in regulating the homeostasis of DP thymocytes independently of MLKL and the necroptotic signaling complex.

While intrinsic apoptosis seem to be the primary pathway regulating steady state thymic selection (197), systemic inflammation due to different triggers such as infections or glucocorticoids leads sudden thymocyte depletion, known as acute thymic atrophy (160, 178, 200). However, it remains unclear whether extrinsic apoptosis by death domain receptors or necrotic cell death modalities such as necroptosis, pyroptosis, or ferroptosis are involved in infection-induced acute thymus atrophy or other types of acute thymus atrophy.

## 4 Age-related thymic involution vs acute thymus atrophy

Despite its crucial role in maintaining immune system homeostasis, the thymus starts undergoing chronological regression from a year of life in a process known as age-related thymic involution (201). Age-related thymic involution is evolutionary conserved across jawed vertebrates (202–205). In humans, the rate of thymus regression is around 3% per year until middle age (35-45 years of age), after which this decreases to a rate of around 1% until death (206). In humans, age-related thymic involution takes place from infancy, but increases after puberty. The most acute phase of age-related thymic involution occurs at 30-40 years in humans and at 9-12 months of age in mice (207). While T cell production regresses throughout life, new naïve T cells can be detected even in individuals of advanced age (208).

Age-related thymic involution is characterized by shrinkage of the thymus, decreased thymus cellularity (18, 209), diminished thymus colonization by TSPs and lower number and proliferation capacity of ETPs (18, 207), reduced number of recent thymic emigrants (reflected by the number of signal joint T-cell excision circles (sjTREC) (210), replacement of lymphoid tissue and fibroblasts by adipose tissue (201, 211), changes in the thymus’s architecture and morphology (209, 212), and defects in the thymic epithelial cell (TECs) compartment (213, 214). Interestingly, memory B cells accumulate in the aging thymus of humans co-localizing with cytokeratin^+^ mTECs (41). Since the thymus cellularity declines with age, the PVS extend to a larger surface in aged individuals (41). As an overall result of age-related thymic involution, a reduced number of recent thymic emigrants can be detected in peripheral blood with age (208, 210). Related to this, no apparent differences in the DP percentage can be observed with age, whereas the decline in thymus cellularity is directly proportional with ETP numbers (215). However, the number of functional TSP/ETP niches does not diminish as we age, rather the pre-thymic progenitors in the blood and bone marrow reduces with age (215), showing that the thymus regression is not merely due to thymic morphologic changes. In addition to the previously described phenomena, the aged thymus exhibit increased reactive oxygen species (ROS), enhanced senescence of TECs and quiescence of TEC progenitors (214) and elevated thymocyte apoptosis (178).

Acute thymic atrophy fundamentally differs from age-related thymic involution (216). Acute thymic atrophy is a stress response and results in reduced thymic cellularity that disproportionally affects developing T cells (216). While age-related thymic involution is a progressive process that worsens throughout life, acute thymic atrophy is a transient state in which the thymus can recover its normal cellularity and tissue architecture. Similarly as age-related thymic involution, acute thymus atrophy is evolutionary conserved in vertebrates (202–205). Acute thymic atrophy can be induced by a wide range of triggers such as infectious diseases (155, 178, 217–223), sepsis (224), and pro-inflammatory cytokines (225, 226).

In this regard, specific pro-inflammatory cytokines have been shown to induce acute thymic atrophy, such as the type 1 cytokines IL-12, IL-18, and IFNγ (31, 227), or the type 2 cytokine IL-33 (225), among others. Duing type 1 immunity, such as infection with the mouse pathogen *Salmonella typhimorium*, ETPs/DN1 fraction remain largly unnaffected during the resultingthymic atrophy (221, 222) while DP thymocytes are espcially sensitive to acute inflammation (200, 221).

Acute thymic atrophy also occurs in response to other triggers, such as malnutrition (228–231), radiotherapy and chemotherapy (232–234), and pregnancy (235–238). Of note, steroid hormones, such as the sex hormones progesterone, androgens, and estrogens (239), as well as glucocorticoids (160, 162) are all capable of inducing thymus atrophy. Pregnancy-induced thymic atrophy is primarily mediated by progesterone, and can be inhibited by the use of progesterone receptor knock out mice (240). Although, steroid hormones, inhibit effector T cell functions (241), thymus atrophy induced by androgens and progesterone is mediated by directly affecting TECs (239). Castration studies revealed that androgens effect on thymic size, appear to be a result of reduced TEC proliferation (242). The steroid hormone progesterone affects TECs by reducing CCL25 production resulting in less ETP recruitment (242). Other studies have also shown that progesterone regulates *Aire* expression in mTECs, thereby affecting negative selection (242, 243).

**Figure 5:**
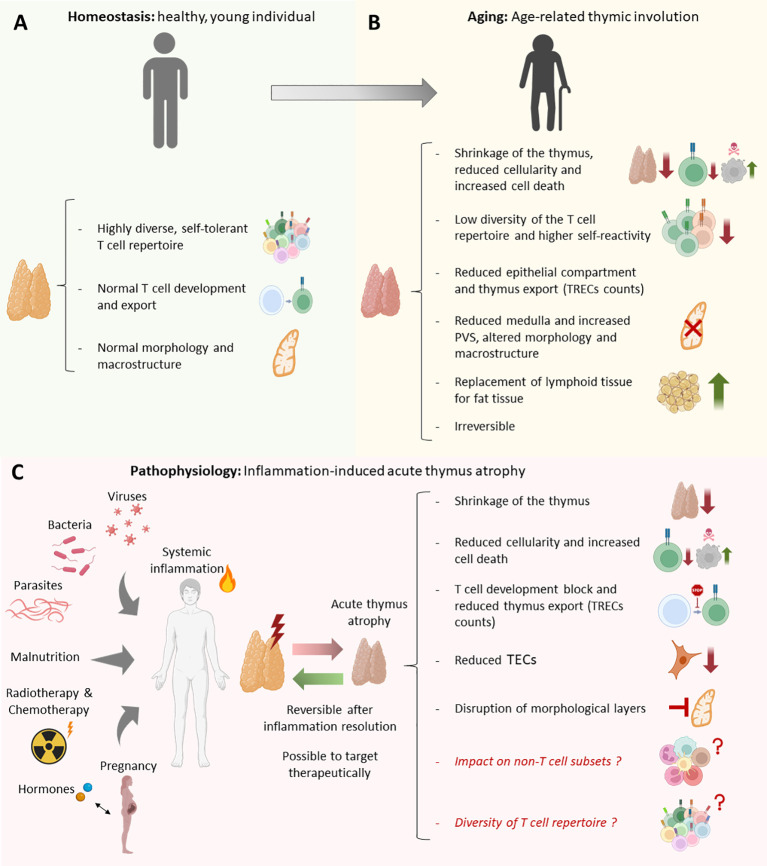
Anatomical, morphological, cellular and molecular differences between the healthy thymus, inflammation induced-acute thymus atrophy, and age-related thymus involution. **(A)** Morphological and cellular characteristics of a young-healthy thymus vs **(B)** aged-individual thymus. **(C)** Common morphological, cellular and molecular alterations produced in inflammation-induced acute thymus atrophy in response to different triggers such as infections, steroid hormones, malnutrition, irradiation, and pregnancy.

The reduction of the thymus cellularity, disorganization of the thymus morphology and shrinkage of the thymus (in size and weight) are the general hallmarks for acute thymic atrophy (216). Interestingly, depending on the initial trigger, there are variations of which stage of T cell development that is blocked and specific morphological changes can be observed (see **Table 2**). The variety of different cytokine and hormonal triggers and their specific thymic effects indicates that that there are different ‘flavors’ of thymus atrophy. However, the different effects these various types of thymus atrophy have on the peripheral immune system remains to be elucidated. Despit various triggers, we have summarised some of the primary features of thymus atrophy (see **Table 2** and **Figure 5**). Systemic inflammation can impact the thymus resulting in acute thymic atrophy.

**Table 2:** Summarized effects of different triggers of acute thymus atrophy on thymus function.

Thymus atrophy trigger	Characteristics of the atrophy	References
Murine cytomegalovirus (MCMV)	0.01% of thymocytes infected Reduce cellularity in the cortex Monocytes and neutrophils infiltration NK cells absence reduce the atrophy ILC1s resistant to damage and increased activation Increased TL1A and IL-18	(31) (36) (219)
Influenza A	Reduced cellularity (loss of DP) Disruption of cortex and medulla distinction Increased IFNγ (by NK cells) NK depletion partly suppress thymic atrophy Thymic DCs contained viral antigens	(217) (244)
*Salmonella typhimurium*	DN1 cells remain unnaffected Reduction of DN2-4 and acute loss of DP thymocytes Release of glucocorticoids and IFNγ that leads to enhance thymocyte death Increase of myeloid cells in the thymus Disruption of the medulla	(221) (222) (245)
Pneumonia Virus of Mice (PVM)	Reduced thymus size and cellularity Reduced numbers of DP and DN4 Increased proportion of myeloid cells Increased levels of TL1A and IL-18	(36)
*Trypanosoma cruzi*	Reduced thymus cellularity (loss of DP) Increase thymocyte egression from the thymus Increase TNFα, IL-17 and IL-6 Increased DN1 and reduced DN2 and DN3 Increased Cell death	(178) (218) (246) (247)
Sepsis (Cecal ligation and puncture, CLP)	Reduced thymus cellularity Disruption of cortex/medulla distinction Increased myelopoiesis and reduced lymphopoiesis Increased extramedullary hematopoiesis	(224)
LPS endotoxin shock	Reduced thymus cellularity Structural disturbance in medulla/cortex distinction Increased proinflammatory cytokines (IFNγ, IL-17, TNFα, IL-6, GM-CSF)	(248)
Irradiation damage	Reduced size and cellularity Reduced developing T cells (DN1-4, DP and SP) and TECs ILCs resistant to damage	(249)
Dexamethasone-induced damage	Reduced size and cellularity Reduced developing T cells and TECs Activation of ILC2s Increased granulocytes, monocytes, macrophages and DCs Increased eosinophils	(33) (32)
Pregnancy-induced atrophy	Reduced numbers of developing T cells Reduced thymus export No changes in cell death (Annexin V) Reduced thymocyte proliferation (BrdU labeling) Reduced numbers of stromal cells	(237, 250)
Rotavirus	Reduced cellularity in C57BL/6J but not in BALB/c or NOD mice Relative increase of macrophages	(251)

Generally, ETPs and DN1 fraction remain unnaffected during *Salmonella typhimorium*-induced thymic atrophy (221, 222), while DP thymocytes have been reported as one of the most sensitive subsets to acute inflammation (200, 221). Remarkably, most of the infectious triggers such as MCMV, Salmonella or *Micobacterium avium* do not specifically target the thymus (219, 221, 252), but it is rather the systemic inflammation that results in the cascade events characteristic of the thymus atrophy. Thus, some cells might be responsile of sensing systemic inflammation may trigger the local dowstream effect in the thymus ultimately leading to atrophy induction. Remarkably, plasma cells were reported to accumulate in the thymus perivascular spaces (PVS) and constitutively secrete immunoglobulin G and show reactivity to common viral proteins (41). Interestingly, beyond the impact of systemic inflammation on the thymus T cell counterpart, some immune subsets are specially resistant to acute inflammation (31, 33, 36, 249, 253). In particular, recent investigations have highlighted the critical role of thymic ILCs in driving the endogenous regeneration of the thymus by producing pro-repair cytokines such as IL-22 (33, 249). Recently, we showed that the type 1 cytokines IL-12 and IL-18, while inducing acute thymus atrophy in mice also enhance the production of neonatal thymic ILC1s, capable of migrating to peripheral organs (31). Additionally, dexamethasone-induced thymus atrophy promoted a type 2 immune response driven by thymic ILC2s, that resulted in tissue regeneration (33).

## 5 Physiological relevance and future perspectives on thymus function, development and atrophy

### 5.1 Physiological implications of the thymus function for human health

The thymus is critical for health and survival of individuals, both in early life and during adulthood. Newborns born with a genetic defect lacking a thymus suffer from a pathology known as severe combined immunodeficiency (SCID), which is lethal in the first years of life unless diagnosed and followed by allogeneic hematopoietic stem cell transplantation (254). Furthermore, a deletion on chromosome 22 results in the failure of thymus development, leading to a pathology known as DiGeorge syndrome (255). DiGeorge syndrome’s clinical manifestations include T-cell deficiency (due to thymic hypoplasia), hypoparathyroidism, cardiac malformations, facial abnormalities neurodevelopmental delay, behavioral, and psychiatric features (255). These patients show higher risk of suffering opportunistic infections (256). Interestingly, patients with partial DiGeorge Syndrome (pDGS) and Down Syndrome (DS) exhibit changes in thymus size (hypoplasia) and architecture, increasing their risk of developing autoimmunity (257). While pDGS patients displayed lower frequency of SP CD4^+,^ SP CD8^+^ and Treg, DS-isolated Tregs showed impaired suppressive capacity (257). These results reveal a link between thymic abnormalities and the immune dysregulation observed in pDGS and DS patients. Myasthenia gravis (MG) is a rare autoimmune disease mediated by antibodies against proteins expressed in the neuro-muscular junction such as the acetylcholine receptor that can be linked to issues with the thymus gland such as increased risk of thymomas (258, 259). The thymus of MG patients shows increased production of inflammatory cytokines and chemokines, leading to B-cell recruitment and the aberrant development of germinal centers in the thymus (259). Thymic B cells secrete antibodies against the acetylcholine receptor. Furthermore, the elevated local inflammation dysregulates the function of thymic Tregs (259). The acetycholine receptor expression in human TECs is elevated by type I and II interferons contributing to a positive feedback loop aggravating the pathology (259). MG patients are frequently subjected to corticosteroid treatment or thymectomy in order to stop thymic B-cell development. Chronic graft-versus-host disease (GVHD) is a complex multiorgan disorder characterized by autoimmunity and immunodeficiency, which arises as a complication of allogeneic hematopoietic cell transplant (HCT) (260). Typically, some months after allogeneic HCT, donor-derived mature T cells cause local inflammation and damage epithelial cells in the skin, liver, and gastrointestinal tract often resulting in sclerosis and fibrosis in multiple organs (261, 262).

A recent study revealed that thymectomized adult patients following cardiac surgery showed increased cancer risk and overall mortality in compared to controls (263). This study confirmed previous reports indicating the importance of the thymus for the maintenance of the immune health in adult individuals (264, 265). Notably, adult individuals are less responsive to vaccination partly due to the age-related thymus involution (266, 267). Despite its importance for the human health, many aspects of thymus biology remain unknown, particularly regarding the cellular and molecular changes during atrophy and the functions of non-T cell subsets.

### 5.2 Future perspectives in thymus development, function and atrophy

Many models of sytemic inflammation leads to acute thymus atrophy heavily impacting the thymus immune landscape. Particularly, the medullary area decreases with age while the perivascular spaces are enlarged (41). However, while the impact of thymic atrophy in the T cell comparment has been thoroughly characterized, the changes in the non-T cell immune compartment, as well as the function of these cells during injury remain elusive. Some recent studies suggest potential roles of non-T cells as sensors of systemic inflammation, initiators of acute thymus atrophy, and conductors of the repair phase post-damage. For example, thymic DCs sense systemic inflammation and can inhibit mTECs proliferation via cell-cell contact in a JAGGED1-NOTCH3 axis dependent manner, ultimately resulting in massive death of developing T cells and acute thymus atropy (268). Notably, B cells are suggested to play a role in the humoral response of the thymus as a protective mechanism against viral infections (41, 269). Macrophages, neutrophils, and eosinophils are proportionally increased during acute thymus atrophy (32, 33, 36, 164). These cells act as phagocytes in response to increased thymocyte death (164, 171). Furthermore, thymus macrophages and neutrophils express high levels of metalloproteases, revealed important in the remodelling of the thymus ECM contributing to a repair response and reestablishment of thymus homeostasis (154, 155, 270). In addition, *MafB^+/GFP^* mice diplayed impaired thymic recovery following irradiation. As *Mafb* expression was mostly restricted to thymus macrophages and monocytes this study illustrates an important role of macrophages and monocytes in thymus repair. The removal of apoptotic cells in the thymus was proven essential to trigger the repair program and the production of BMP-4, IL-23, IL-22 by the stroma to restore thymus homeostasis (271).

ILCs produce cytokines that can alter the development and fate of developing T cells. Group-1 ILCs are relatively increased upon viral infection (31, 244), proinflammatory cytokine exposure (31), or following irradiation (226), resulting in increased production of IFN-γ and Perforin, among others (31, 244). These factors have been shown to drive acute thymus atrophy as unveiled in *Ifnγ* ^-/-^ (244) and *Prf1^-/-^* (226) mouse models. Additionally, targeting of NK1.1^+^ cells (majority of group-1 ILCs in the thymus) ameliorated irradiation induced-acute thymus atrophy (226). Notably, ILC2s were also relatively increased and activated in dexamethasone-induced thymus atrophy (33). Dexamethasone - activated ILC2s increased the expression of genes such as *Csf2*, *Il13*, *Il5*, or *Areg* known to impact thymus stroma, myeloid compartment and mTEC proliferation. In line with these findings, a study conducted in 2012, revealed that following irradiation, thymic DCs produce IL-23 leading to IL-22 secretion by thymus LTis resulting in enhance TECs proliferation and survival (249). Studies identifying the critical mediators responsible for inducing thymus atrophy and promoting thymus regeneration are crucial for understanding the most effective approaches to enhance thymus function. In the past few years, BMP4 was identified as a potent endogenous thymus regeneration factor by enhancing *Foxn1* expression in TECs (253). These studies offer innovative strategies to achieve thymus rejuvenation.

In fact, some studies have shown that there are treatments that enhance thymus function and output. The treatment with ghrelin enhance the production and development of T cells and promotes the engraftment of T cells in SCID mice (146). Similarly, IL-7 plays a key, non-redundant role in the generation of the T-cell repertoire (272), and its administration enhances T cell development (273). Linked to this, a recent study has shown that CAR-T cell transplantation cultured with IL-7 and IL-15 improve the efficiency of the transplantation and reduced the exhaustion of the transplanted cells (274).

Altogether, non-T cell immune subsets play a crucial role in maintaining T cell homeostasis and creating the proper environment for T cell development. Simultaneously, they also play key roles in initiating acute thymus atrophy and facilitating repair processes after damage, thus restoring homeostasis. This may occur through various mechanisms such as (1) the release of proinflammatory cytokines, (2) direct cell-cell interactions with thymic epithelial cells (TECs) and thymocytes leading to cell death in neighboring cells, or (3) actively inducing morphological changes in thymus architecture through ECM remodeling, resulting in increased thymocyte death and developmental arrest.

Since the availability of human thymus samples is low, murine studies to investigate thymus changes induced by systemic inflammation are very relevant as, conforming a limitation for performing functional studies in humans. Instead, a recent study utilized spatial omics techniques in the human thymus (275). These emerging technologies including spatial transcriptomics resolution, combined with mouse genetic studies hold promise for elucidating the interactions between developing T cells and the stroma, potentially providing insights for interventions to either promote or inhibit T cell development according to disease requirements. Another promising alternative is the use of tissue-engineered artificial human thymus from human iPSCs (276), or the recently generated thymus organoids (277). These models may better resemble the physiological conditions in humans and help determine the extent to which findings from murine studies are applicable to humans.

Looking forward, it remains unclear whether extrinsic apoptosis by death domain receptors and necrotic cell death modalities such as necroptosis, pyroptosis, or ferroptosis are involved in acute thymus atrophy. Additionally, optimizing thymus function through the administration of cytokines or hormones to inhibit thymus atrophy may prove beneficial in certain immunotherapies against cancer, infectious diseases, or autoimmunity. However, further research into the various aspects of thymus atrophy and its regeneration are required understand when it would be most beneficial to support thymic T cell development.

## Glossary

Ach: Acetylcholine
AGM: Aorta-gonad mesonephros
APC: Antigen presenting cell
APCs: Antigen-presenting cells
aSMase: Acidic Sphingomyelinase
BM: Bone Marrow
CAR-T: Chimeric Antigen Receptor T-cell
CD: Cluster of Differentiation
CDPs: Common Dendritic Progenitors
CLP: Cecal Ligation and Puncture
CLP: Common-lymphoid progenitor
CLP-2: Common Lymphoid Progenitors-2
CMJ: Corticomedullary junction
CMP: Common-Myeloid Progenitor
CNS: Conserved non-coding sequence
cTEC: Cortical thymic epithelial cell
CTP: Circulating T cell progenitor
DCs: Dendritic cells
Dex: Dexamethasone
Dll4: Delta-like 4
DN: Double negative
DP: Double positive
DR3: Death Receptor 3
DR5: Death Receptor 5
DS: Down syndrome
E#: Embryonic day
ECM: Extracellular Matrix
ELPs: Early Lymphoid Progenitors
ETP: Early Thymic Progenitor
FADD: FAS-Associated Protein with Death Domain
FOXP3: Forkhead box P3
GALT: Gut-associated lymphoid tissue
GH: Growth Hormone
GMP: Granulocyte-monocyte progenitor
GPCR: G protein-coupled receptor
GVHD: Graft-Versus-Host Disease
HCT: Hematopoietic cell transplant
HSC: Hematopoietic stem cell
IEL: Intestinal intraepithelial lymphocyte
ILC: Innate lymphoid cell
iNKT: Invariant natural killer T cell
IPEX: Immune dysregulation, polyendocrinopathy, enteropathy, X-linked syndrome
iPSC: Induced Pluripotent Stem Cell
LFA-1: Lymphocyte Function-Associated Antigen 1
LMPP: Lympho-myeloid primed progenitor
LPS: Lipopolysaccharide
LSK: Lineage-Sca-1^+^ c-Kit^+^
MAPK: Mitogen-Activated Protein Kinase
MCMV: Murine Cytomegalovirus
MDP: Macrophage-Dendritic Progenitor
MG: Myasthenia gravis
MHC-I: Major histocompatibility complex I
MHC-II: Major histocompatibility complex II
MLKL: mixed lineage kinase domain like
MPP: Multipotent progenitor
mTEC: Medullary thymic epithelial cell
NE: Norepinephrine
NK: Natural killer
OT: Oxytocin
pDGS: Partial DiGeorge syndrome
PCD: Programmed cell death
pDC: Plasmacytoid dendritic cell
PLC: Phosphoinositide-Specific Phospholipase C
PVM: Pneumonia Virus of Mice
PVS: Perivascular spaces
RIPK1: Receptor-Interacting Protein Kinase 1
RIPK3: Receptor-Interacting Protein Kinase 3
ROS: Reactive Oxygen Species
SCF: Stem Cell Factor
SCID: Severe Combined Immunodeficiency
sjTREC: Signal Joint T-Cell Receptor Excision Circle
SLC: Solute carrier transporter
SP: Single positive
T3: Triiodothyronine
T4: Thyroxine
TCR: T cell receptor
THF: Thymic Humoral Factor
TL1A: TNF-like ligand 1A
Treg: Regulatory T cell
TSLP: Thymic stromal lymphopoietin
TSP: Thymus seeding progenitor
VLA: Very Late Antigen
VP: Vasopressin
WNK1: With no lysine kinase 1
